# Piezoelectric Effect and Electroactive Phase Nucleation in Self-Standing Films of Unpoled PVDF Nanocomposite Films

**DOI:** 10.3390/nano8090743

**Published:** 2018-09-19

**Authors:** Marco Fortunato, Chandrakanth Reddy Chandraiahgari, Giovanni De Bellis, Paolo Ballirano, Francesca Sarto, Alessio Tamburrano, Maria Sabrina Sarto

**Affiliations:** 1 Department of Astronautical, Electrical and Energy Engineering, Sapienza University of Rome, Via Eudossiana 18, 00184 Rome, Italy; giovanni.debellis@uniroma1.it (G.D.B.); Alessio.tamburrano@uniroma1.it (A.T.); mariasabrina.sarto@uniroma1.it (M.S.S.); 2 Research Center for Nanotechnology Applied to Engineering of Sapienza (CNIS), SNNLab, Sapienza University of Rome, Piazzale Aldo Moro, 5, 00185 Rome, Italy; Paolo.ballirano@uniroma1.it; 3 Department of Physics, Indian Institute of Technology Delhi, New Delhi 110016, India; chandra.nano@gmail.com; 4 Department of Earth Sciences, Sapienza University of Rome, Piazzale Aldo Moro 5, 00185 Rome, Italy; 5 ENEA, Frascati Research Center, Via Enrico Fermi, 45, Frascati, 00044 Rome, Italy; francesca.sarto@enea.it

**Keywords:** polyvinylidene fluoride nanocomposite, piezoelectric effect, piezoresponse force microscopy

## Abstract

Novel polymer-based piezoelectric nanocomposites with enhanced electromechanical properties open new opportunities for the development of wearable energy harvesters and sensors. This paper investigates how the dissolution of different types of hexahydrate metal salts affects *β*-phase content and piezoelectric response (d_33_) at nano- and macroscales of polyvinylidene fluoride (PVDF) nanocomposite films. The strongest enhancement of the piezoresponse is observed in PVDF nanocomposites processed with Mg(NO_3_)_2_⋅6H_2_O. The increased piezoresponse is attributed to the synergistic effect of the dipole moment associated with the nucleation of the electroactive phase and with the electrostatic interaction between the CF_2_ group of PVDF and the dissolved salt through hydrogen bonding. The combination of nanofillers like graphene nanoplatelets or zinc oxide nanorods with the hexahydrate salt dissolution in PVDF results in a dramatic reduction of d_33_, because the nanofiller assumes a competitive role with respect to H-bond formation between PVDF and the dissolved metal salt. The measured peak value of d_33_ reaches the local value of 13.49 pm/V, with an average of 8.88 pm/V over an area of 1 cm^2^. The proposed selection of metal salt enables low-cost production of piezoelectric PVDF nanocomposite films, without electrical poling or mechanical stretching, offering new opportunities for the development of devices for energy harvesting and wearable sensors.

## 1. Introduction

During the last decade, flexible piezoelectric films have been attracting a great deal of interest for the realization of devices capable of converting low-frequency mechanical energy into electrical signals. One of the most interesting flexible piezoelectric organic materials is polyvinylidene fluoride (PVDF), which is a semicrystalline polymer with excellent piezoelectric and pyroelectric characteristics. PVDF can be obtained in three main polymorphs, namely, *α*, *β*, and *γ* forms, giving it a large number of engineering applications, spanning from capacitors to sensors and actuators [1]. The *β* and *γ* are the only phases displaying pyroelectric and piezoelectric properties [2]. The increase of the *β*-phase content is considered an essential prerequisite to the enhancement of the piezoelectric response of PVDF polymer thin films [3,4].

A conventional way to induce a preferred orientation of the dipoles along the field direction, thus increasing the electroactive response of the material, is electric field poling, which consists of applying a very high DC electric field (in the range of 10^6^ V/m) to the sample at elevated temperatures (around 120 °C) [5]. However, electric poling is not a convenient or cost-effective industrial approach. Recently, in order to overcome this problem associated with the nucleation of the electroactive state in PVDF, alternative strategies, such as mechanical stretching [6,7]; heat-controlled spin coating [8]; addition to the PVDF matrix of external additives such as clay [9], metal oxides [10,11], metal nanoparticles [12,13], or ceramic filler [14]; or a combination of spin coating and additive dissolution [15] have been proposed. Such additives are said to yield a large increase in the *β*-phase content. Several experimental and theoretical studies have confirmed that the use of carbon nanotubes (CNTs) as filler in the PVDF matrix can lead to a relevant increase in the *β*-phase content [16,17]. Moreover, the role of graphene nanoplatelets (GNPs) in the nucleation of the electroactive phase in PVDF nanocomposites has been recently investigated [18,19]. GNPs are 2D nanostructures having a high aspect ratio and a large surface area, which promotes a very strong interfacial interaction with the polymeric chains in nanocomposites. This results in an enhancement of the electric [20], mechanical [18], piezoresistive [21], and piezoelectric [22] properties of the host polymer.

The use of ZnO nanorods as fillers in PVDF nanocomposites has been also proposed [23,24], with the aim of exploiting the synergistic effect on the piezoresponse of piezoelectric ZnO nanostructures [25,26] and PVDF.

An alternative method to enhance the piezoelectric phase in PVDF is the dissolution of a hexahydrate metallic salt (HMS) during the production process of the polymeric film. It was demonstrated that the hydrogen bonding interaction between HMS and PVDF contributes to the *β*-phase nucleation. This effect was observed using Ce or Y nitrate hexahydrate [27] or Mg chlorate hexahydrate [28]. However, in these studies, the direct correlation between the increase of the piezoelectric phase fraction and the corresponding increase of the piezoresponse coefficient (i.e., *d*_33_), which is a crucial parameter describing the amplitude of the displacement inside the material per unit voltage, is not discussed. Within this context, the local characterization at nanoscale through piezoresponse force microscopy (PFM) of the piezoelectric properties of modified PVDF [29,30] is a crucial step towards the optimization of material production and device performance.

In our previous works we have investigated the influence of GNP dispersion in PVDF nanocomposites on both *β*-phase formation [18] and *d*_33_ enhancement through PFM [22]. In one case, we found a *d*_33_ of GNP–PVDF nanocomposites limited to 5.2 pm/V. In a further study, we proposed two different GNP dispersion routes and the addition of ZnO NRs to PVDF as fillers [23], thus obtaining a *d*_33_ of ~6.2 pm/V, with a fraction of the *β*-phase (F(β)) below 64%.

Higher values of F(β) have been obtained in HMS PVDF nanocomposite films. In particular, using cerium(III)/yttrium(III) nitrate hexahydrate salts (Ce(NO_3_)_3_·6H_2_O and Y(NO_3_)_3_·6H_2_O), which are well known to be toxic and highly expensive salts, an F(β) value as high as 82% has been reported [27]. In [28], it was shown that the dissolution of 0.2 wt % magnesium chloride hexahydrate (MgCl_2_·6H_2_O), which is a nontoxic, cost-effective salt, was enough to achieve a *β*-phase fraction well beyond 80%. However, the production process is very time consuming because it requires as long as 2 days of magnetic stirring of the PVDF–HMS mixture. Nevertheless, in both studies, the amount of F(β) was not correlated to the local or global value of *d*_33_.

In this paper, we investigated the use of different HMSs dissolved in PVDF with the aim of maximizing both the *β*-phase enhancement and the piezoresponse coefficient *d*_33_ at nano- and microscales of the resulting nanocomposite films. Moreover, we analyzed the correlation between surface morphology of the produced samples (i.e., the spherulite average diameter) and piezoelectric properties.

It is demonstrated that the highest enhancement of F(β) and of *d*_33_ (up to 82% and ~13.49 pm/V as peak value, respectively) is obtained in PVDF nanocomposite films modified with Mg nitrate hexahydrate, corresponding to a minimum of the spherulite diameter. In addition, among the investigated HMSs, the worst piezoelectric performance was observed in the PVDF nanocomposite containing the Fe salt. The combined effect of HMS-dissolution and addition of a nanofiller was also investigated.

Finally, the developed production route of piezoelectric PVDF nanocomposite film is fast and cost effective because it requires only 3 h of magnetic stirring (against days of processing) and makes use of inexpensive and low-toxicity commercially available hexahydrate salts of magnesium.

## 2. Materials and Methods

PVDF self-standing nanocomposite films were produced using PVDF with molecular weight 300.000–330.000 g/mol (Solef 6010, Solvay Specialty Polymers S.P.A., Bollate, Italy). Graphene nanoplatelets (GNPs) were obtained through liquid-phase exfoliation by probe sonication of commercially available Graphite Intercalation Compound (GIC) (GrafTech International Holdings Inc., Brooklyn Heights, OH, USA), thermally expanded at 1150 °C for 5 s [20,31]. ZnO NRs were produced through the thermal decomposition method [25]. N,N dimethylformamide (DMF ≥99%, Sigma–Aldrich, Saint Louis, MO, USA), acetone (ACS reagent, ≥99.5%, Hampton, NH, USA), zinc nitrate hexahydrate [Zn(NO_3_)_2_·6H_2_O] (≥98%, Sigma–Aldrich, Saint Louis, MO, USA), magnesium nitrate hexahydrate [Mg(NO_3_)_2_·6H_2_O] (Sigma–Aldrich, ACS reagent, 99%), magnesium chloride hexahydrate [MgCl_2_·6H_2_O] (Sigma–Aldrich, ≥99%), aluminum chloride hexahydrate [AlCl_3_·6H_2_O] (Sigma–Aldrich, ≥99%), and iron chloride hexahydrate [FeCl_3_·6H_2_O] (Sigma–Aldrich, ≥97%) were used as received.

### 2.1. Preparation Process of PVDF-Based Polymers

Neat PVDF films were prepared using the procedure described in [23,24]. Briefly, 5 wt % PVDF powder was dissolved in 20 mL of a solvent mixture consisting of DMF and acetone (1:1 v/v), chosen according to Hansen’s solubility parameters as a good combination to fully dissolve PVDF [32]. A clear and transparent solution was obtained upon continuous stirring at room temperature for 3 h, ensuring the complete dissolution of PVDF. In order to have a complete evaporation of the solvent, the solution was casted onto a clean glass plate and placed in an oven at 100 °C for 12 h. Finally, the obtained films, having thickness of approximately 15 μm each, were peeled off the substrate (sample Neat-PVDF in Table 1).

**Figure 1:**
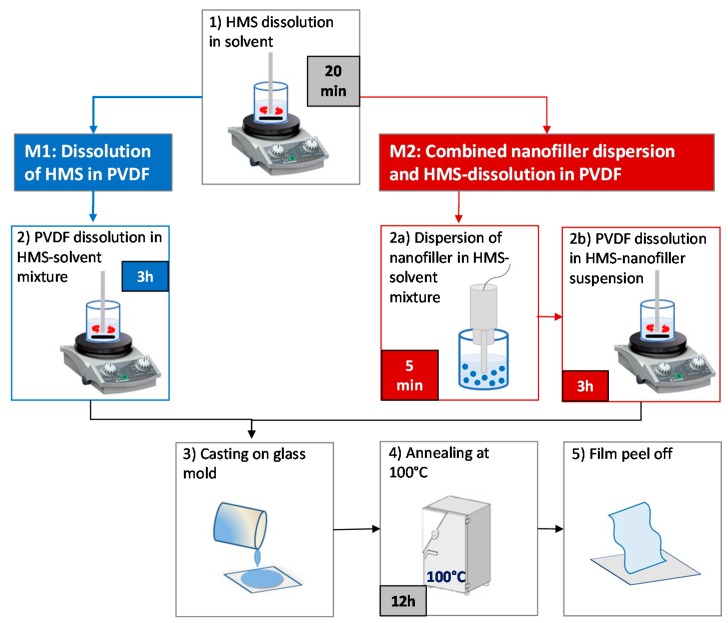
Schematic illustration of the solution-derived PVDF nanocomposite thin film preparation process.

The PVDF films produced with the addition of 0.2 wt % hexahydrate salts of different metals (like zinc, manganese, aluminum, iron) were prepared according to [24]. The samples were prepared through dissolution of the HMS in a solvent mixture of DMF and acetone (1:1 v/v) (Figure 1, M1). Next, PVDF powder was added to the as-obtained nanofiller suspension and stirred for 3 h. As sketched in Figure 1, M1, upon casting the obtained solution on a clean glass plate and evaporating the solvent at 100 °C for 12 h, we obtained a flexible self-standing film. The HMS concentration was chosen based on preliminary FTIR measurements carried out in a previous study as the one corresponding to the most intense peaks of the *β*-phase in the IR spectrum of PVDF nanocomposite [24].

In order to produce PVDF self-standing nanocomposite films filled with GNPs or ZnO NRs, at first, HMS was dissolved in a solvent mixture of DMF and acetone (1:1 v/v). Next, the nanofiller (either GNPs or ZnO NRs, 0.1 wt %) was dispersed homogeneously in the HMS solution through a short probe sonication (5 min in pulse mode at 40% in power amplitude). Finally, PVDF powder was added to the as-obtained colloidal suspension and stirred for 3 h, and the nanocomposite films were finally obtained as described above and sketched in Figure 1, M2.

### 2.2. Characterization

A Field Emission Scanning Electron Microscope (FE-SEM, Auriga, Carl Zeiss, Oberkochen, Germany) operating with an accelerating voltage of 3 kV was used to assess the morphology of the PVDF nanocomposite films. A Quorum Technologies Q150T ES sputter coater (Laughton, East Sussex, UK) was used to metallize the PVDF films prior to SEM imaging with 20 nm of Cr, in order to prevent charging.

FT-IR measurements were performed using the same setup described in our previous work [22,23]. FT-IR measurements were carried out in the 4000–600 cm^−1^ range with a resolution of 1 cm^−1^.

X-ray powder diffraction (XRPD) measurements were performed using the same instrument and the same procedure described in [26]. Briefly, we used Cu *Kα* radiation (*λ* = 0.15418 nm) operating at 40 kV and 40 mA and operating in transmission mode. Data were collected in a 2θ angular range extending from 7° to 100° with a step size of 0.022° and 1 s counting time. Samples were prepared as capillaries loaded with nanostructures in powder form, obtained after three steps of dispersion using a high-shear mixer.

### 2.3. Piezoelectric Response Measurement

**Figure 2:**
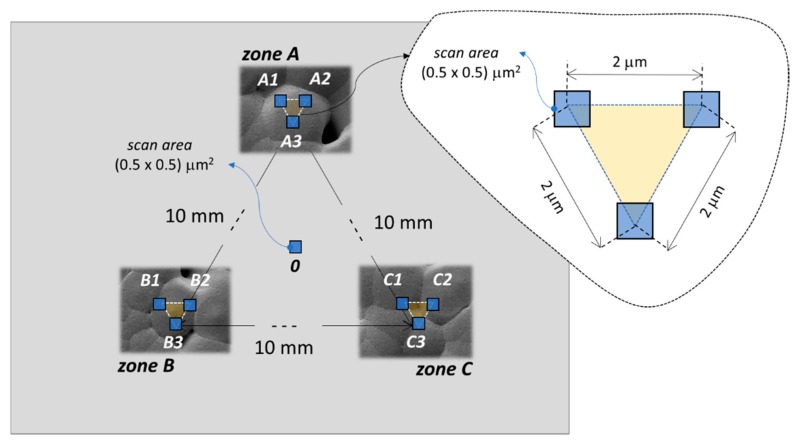
Sketch of the measurement points for the local piezoresponse of the sample through piezoresponse force microscopy (PFM).

The piezoelectric properties of our samples were assessed through PFM [29] measuring the piezoelectric coefficient $d_{33}$. For this purpose, we used a commercial Bruker-Veeco Dimension Icon AFM (Billerica, MA, USA) with a Co–Cr-coated-tip silicon cantilever (MESP-RC-V2, Bruker, Billerica, MA, USA) [22]. We applied to the tip an alternating voltage with the frequency of 15 kHz, and an increasing maximum amplitude *V_ac_* of 2, 4, 6, 8, or 10 V. The bottom electrode of the samples was grounded. We scanned 10 different areas (500 × 500) nm^2^ in size, with 256 × 256 acquisition points per scanning area, and with a scan rate of 0.5 Hz. The 10 scanning areas were located in four different zones of the sample surface, as shown in Figure 2. The first scanning area (labeled “0” in Figure 2) was located in the center of the sample, and was used as an approach area needed to verify whether the sample had a piezoelectric response. The next 9 scanning areas were located in three different zones of the sample surface, 10 mm apart from each other, as shown in Figure 2. Two out of the three zones (Zone A and Zone B) are selected in proximity of the center of two different spherulites, whereas the third one includes the valley between two adjacent spherulites. This choice was made in order to characterize the piezoresponse of areas of the sample with different morphologies and characteristics, with the aim of getting information about the uniformity of the piezoresponse over the whole sample surface.

The procedure we applied to measure the piezoresponse of the sample through PFM includes the following steps. First, we measured a calibration sample (Bruker SKU:PFM-SMPL, Billerica, MA, USA) constituted of a periodically poled lithium niobate (PPLN) having a nominal piezoelectric coefficient of $d_{33,\mathrm{PPLN}}=7.5$ pm/V. In this case, the scan area was (60 × 7.5) μm^2^ in size with (256 × 32) measured points, the scan rate was 0.5 Hz, and the applied 15 kHz alternating voltage had increasing amplitude of 2, 4, 6, 8, or 10 V. The amplitude of the PFM signal $\left(V_{piezo}\right)$ resulting from the average of the (256 × 32) measurement points over the scanning area is given by [33]

(1) $$V_{piezo}=\xi \;d_{33,PPLN}\;V_{ac}$$

in which ξ is the calibration parameter that allows conversion of $V_{piezo}$ (expressed in mV) into the vertical displacement ($A_{piezo}$, expressed in pm). The calibration factor ξ is given by the ratio of the slope ($m_{PPLN}$) of the linear fit of the PFM signal $V_{piezo}$ to the amplitude of the applied voltage ($V_{ac}$), and the nominal piezoelectric coefficient of the reference sample $\left(d_{33,\mathrm{PPLN}}\right)$:

(2) $$\xi =\frac{m_{PPLN}}{d_{33,\;\mathrm{PPLN}}}\;.$$

Once ξ was obtained from Equation (2), we measured the PFM signal of a PVDF nanocomposite sample. For this purpose, we first scanned the approaching area (0 in Figure 2) and then the three scanning areas in each selected zone (A, B, C in Figure 2). For each *i*th scanning area, we obtained the displacement $A_{piezo}^{i}$ averaged over the (256 × 256) measurement points as follows:

(3) $$A_{piezo}^{i}=V_{piezo}^{i}/\xi .$$

Since $A_{piezo}^{i}$ is also related to the applied voltage by [29,33]

(4) $$A_{piezo}^{i}=d_{33}^{i}V_{ac}$$

we obtain the piezoelectric coefficient $d_{33}^{i}$ of the *i*th scanning area as the slope of the linear fit of $A_{piezo}^{i}$ versus the applied voltage $V_{ac}$. The piezoresponse coefficient of the *k*th zone of the sample ($d_{33}^{\mathrm{zone}\;k}$), is then computed as the average value of the coefficients of the three scanned areas in that zone:

(5) $$d_{33}^{\mathrm{zone}\;k}=\overset{3}{\underset{i=1}{\sum }}d_{33}^{i}/3.$$

After completing the PFM characterization of the considered PVDF sample, the reference PPLN was tested again in order to verify that the system was still calibrated. For this purpose, we repeated the measurement of the piezoelectric signal ($V_{piezo}$) of the reference PPLN sample and we compared the new value with the corresponding value previously measured (before the characterization of the PVDF sample). If the relative error between the two piezoelectric signals for each value of the applied voltage $V_{ac}$ was less than 20%, the measurement of the PVDF sample was considered valid and calibrated [29].

Once the PFM measurements were performed in each selected zone of the sample, we estimated the average PFM response as

(6) $$\langle d_{33}\rangle =\left(d_{33}^{A}+d_{33}^{B}+d_{33}^{C}\right)/3.$$

The standard deviation of $\langle d_{33}\rangle$ is representative of the uniformity of the piezoresponse of the sample.

Finally, in order to assess the proposed procedure, we contacted one of the produced PVDF samples (M1-HS2 in Table 2) with a top and a bottom gold electrode, and we measured the global piezoelectric coefficient of the sample using a commercial mini-shaker (Sinocera, YE2730A, Yangzhou City, Jiangsu Province, China) operating with an amplitude force of 0.25 N and with a frequency of 110 Hz.

## 3. Results and Discussion

PVDF nanocomposite films were produced via a solution processing method as described in the experimental section above, using different types of HMSs eventually combined with a nanofiller (like GNPs or ZnO NRs) in order to enhance the piezoelectric response of the film. Two different methods were developed in order to produce large-scale PVDF film through the dissolution of HMS in PVDF (Method M1) or by combining HMS-dissolution and nanofiller dispersion (Method M2).

The complete list of the produced samples is reported in Table 1.

### 3.1. Surface Morphology

The surface morphology of PVDF nanocomposite samples was analyzed through FE-SEM. The surface of the PVDF films was characterized by a spherulitic structure. The diameter of the spherulites was evaluated from FE-SEM images using a commercial image processing software (ImageJ ©, National Institute of Health, Bethesda, MD, USA). The mean value of the spherulite diameter was estimated by averaging the diameters of 10 different spherulites. The obtained values are reported in Table 2. It is worth noting that the spherulite diameter is significantly influenced by the interaction between the metallic ions in the HMS and the CF_2_ group of PVDF.

**Figure 3:**
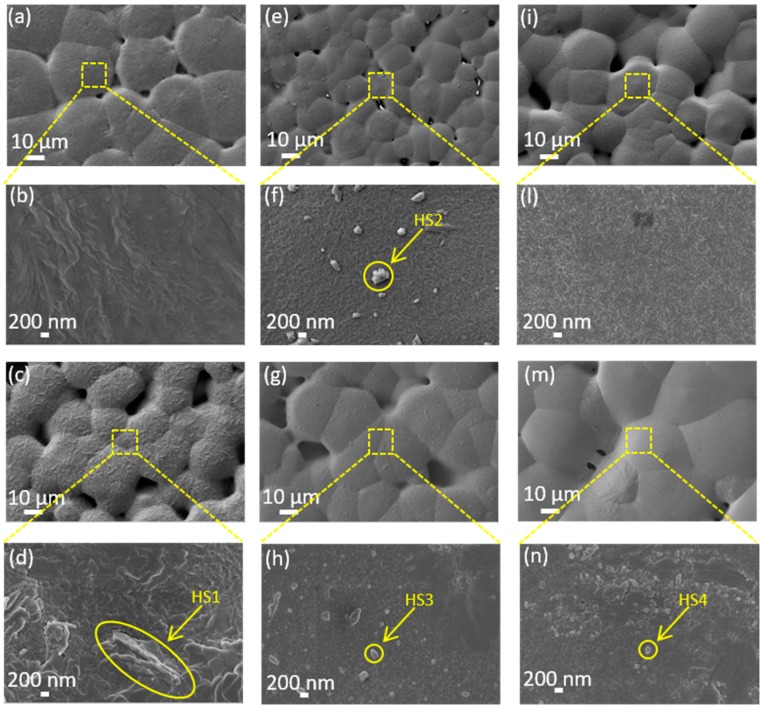
FE-SEM low-magnification and high-magnification micrographs of (**a**,**b**) Neat-PVDF, (**c**,**d**) M1-HS1 film, (**e**,**f**) M1-HS2 film, (**g**,**h**) M1-HS3 film, (**i**,**l**) M1-HS4 film, and (**m**,**n**) M1-HS5 film.

**Figure 4:**
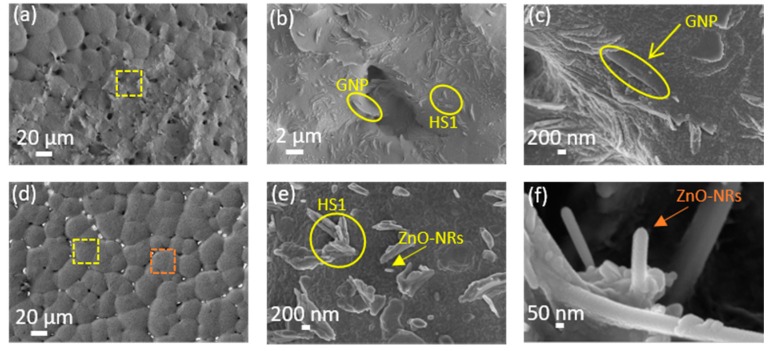
FE-SEM low-magnification and high-magnification micrographs of the sample M2-HS1-GNP (**a**–**c**), and of the sample M2-HS1-ZnO (**d**–**f**).

Figure 3 and Figure 4 show the FE-SEM micrographs at low and high magnifications of neat-PVDF, and of samples from Table 1 prepared with the addition of HMS or HMS plus nanofillers (either GNPs or ZnO NRs). The typical spherulitic morphology is largely affected by the nucleation and formation of the polymer chains during the solid–liquid phase separation. The yellow arrows in Figure 3 and Figure 4 show HMS crystals or nanofillers distributed within the polymer matrix. The interaction between HMS and polymer modifies the morphology of the composite, in particular affecting the average spherulite diameter. In general, we observed a reduction of the spherulite diameter upon addition of the HMS, except for FeCl_3_·6H_2_O. HMS originates nucleation sites in the polymer, owing to the strong interfacial interaction between the metallic ion of the HMS and the polymeric chain. On the other hand, the combination of both HMS and nanofillers dispersed inside the polymer does not induce a reduction of the spherulite diameters.

### 3.2. Electroactive β-Phase Enhancement

FT-IR spectroscopy and XRPD were employed to verify the presence of the *β*-phase in PVDF nanocomposite films.

#### 3.2.1. FT-IR Analysis

As is well known from the literature [12,25,34], the characteristic FT-IR peaks of the *α*-phase are located in the range between 1423 and 763 cm^−1^, those of the *β*-phase at 1275 cm^−1^ and 840 cm^−1^, and those of the *γ*-phase at 1234 cm^−1^.

**Figure 5:**
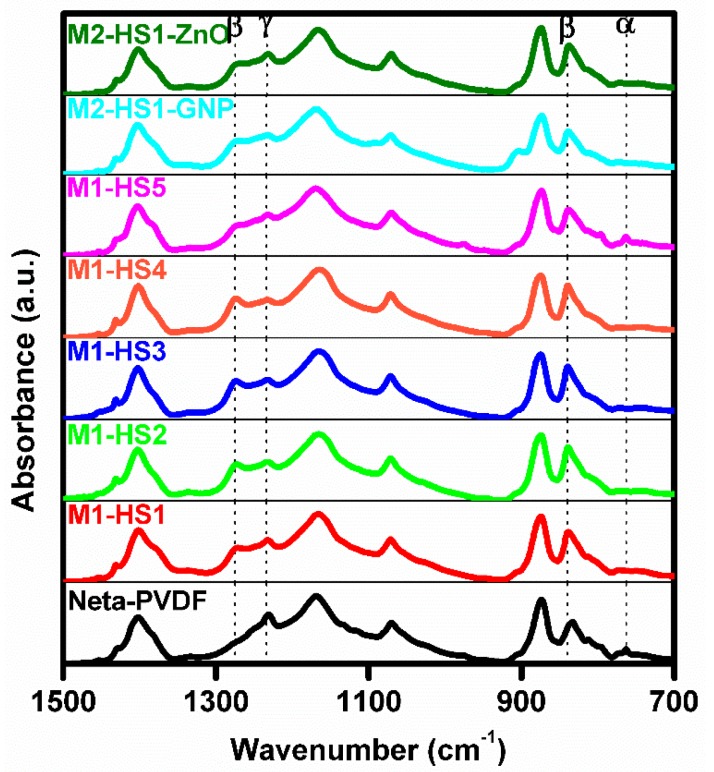
FT-IR spectra of PVDF nanocomposite samples.

Figure 5 shows that all of the samples of Table 1, produced following either Method M1 or M2, exhibit the presence of the *β*-phase, as highlighted from the characteristic peak at 840 cm^−1^. According to [35], the relative volume of the *β*-phase fraction F(β) of the produced samples can be estimated from the values $A_{\alpha }$ and $A_{\beta }$ of the absorbance at the wavelengths (766 and 840 cm^−1^) associated to the main peaks of the *α*- and *β*-phases, respectively, using the following formula:

(7) $$F\left(\beta \right)=\frac{A_{\beta }}{\left(K_{\beta }/K_{\alpha }\right)A_{\alpha }+A_{\beta }}$$

in which it is assumed that the ratio between the absorption coefficients of the *α*- and *β*-phases is $K_{\beta }/K_{\alpha }\sim 1.3$.

The obtained values of F(β) are reported in Table 2. We notice that in all produced samples of PVDF nanocomposite, apart from M1-HS5, the computed value of F(β) is higher than in the plain PVDF sample. The highest value of F(β) ~ 82% was obtained for the M1-HS2 sample, which was produced by dissolving Mg nitrate hexahydrate in PVDF. This sample is also characterized by the smallest spherulite diameter of ~12 μm on average (Table 2). The obtained values are comparable to those reported in literature for PVDF nanocomposites obtained using hexahydrate salts of rare earths, like Ce nitrate hexahydrate and Y nitrate hexahydrate [27].

The reason for the lower amount of *β*-phase observed in the PVDF sample with added FeCl_3_·6H_2_O with respect to the other samples can be ascribed to the relatively high mass and low negative standard redox potential of Fe^3+^ (Fe^3+^ + 3e ⇌ Fe, −0.037 eV), which weaken H-bond formation between PVDF chains and hexahydrate salts in polar solvents. Consequently, nucleation of the *β*-phase in the PVDF nanocomposite is limited. On the contrary, the best result obtained with the Mg nitrate hexahydrate is attributed to the highly negative standard redox potential of magnesium (Mg^2+^ + 2e ⇌ Mg, −2.373 eV), which is the same as that of yttrium (Y^3+^ + 3e ⇌ Y, −2.372 eV) and very close to that of cerium Ce^3+^ (Ce^3+^ + 3e ⇌ Ce, −2.336 eV) [36].

#### 3.2.2. XRPD Analysis

**Figure 6:**
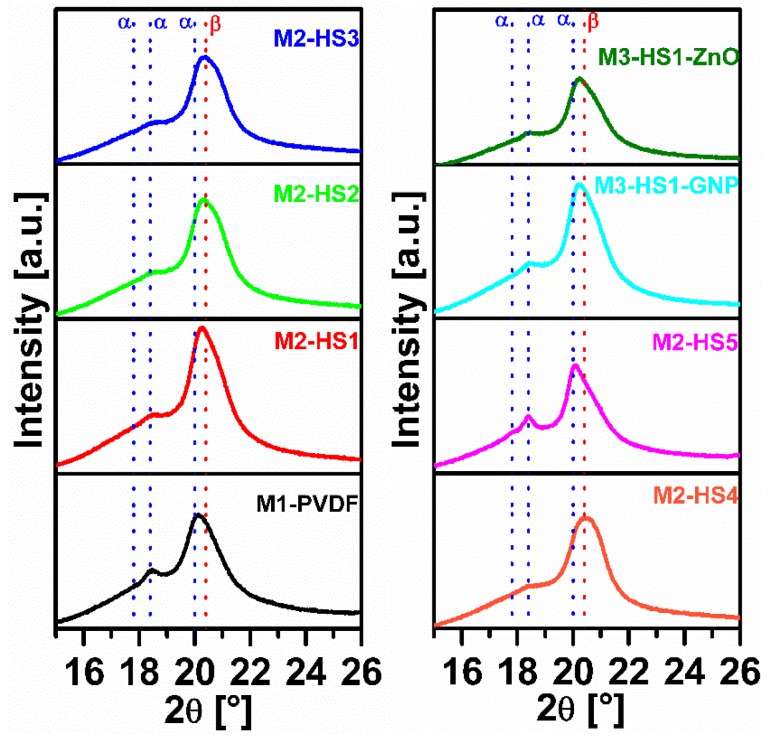
X-ray powder diffraction (XRPD) patterns acquired on the produced samples of PVDF nanocomposites.

Figure 6 shows the XRPD pattern of the produced samples. An easy discrimination and quantification of the *α*- and *β*-phases can be devised from the intensity of the two relatively strong (100) and (020) reflections, located at ca. 17.8° and 18.4° 2θ, that are typical of the *α*-phase [18]. Accordingly, we observe that the *α*-phase is the most abundant one in the neat PVDF sample. When we add the HMS, we observe a partial reduction of the intensity of the two (100) and (020) reflections, and a broadening and shift of the position of the strongest peak from 20° to 20.4° [18]. This peak results more from the coalescence of the strong (110) reflection of both phases than from the *β*-phase being located at a slightly higher angle. This behavior is less evident in the sample prepared with the addition of the Fe-HMS (M1-HS5) and in the samples containing HMS plus nanofillers. Therefore, it is confirmed that the occurrence of *β*-phase is prevailing in the samples with the HMS (apart from M1-HS5) with respect to the samples containing both HMS and nanofillers. Based on the XRPD and FT-IR data, we can conclude that a very small amount of HMS (0.2 wt %) hinders the *α*-phase nucleation and preferentially promotes the polar *β*-phase formation. A possible mechanism for the *β*-phase enhancement induced by the dissolved HMS is the hydrogen bonding interactions between HMS and the CF_2_ groups of PVDF [28].

### 3.3. Piezoelectric Effect

**Figure 7:**
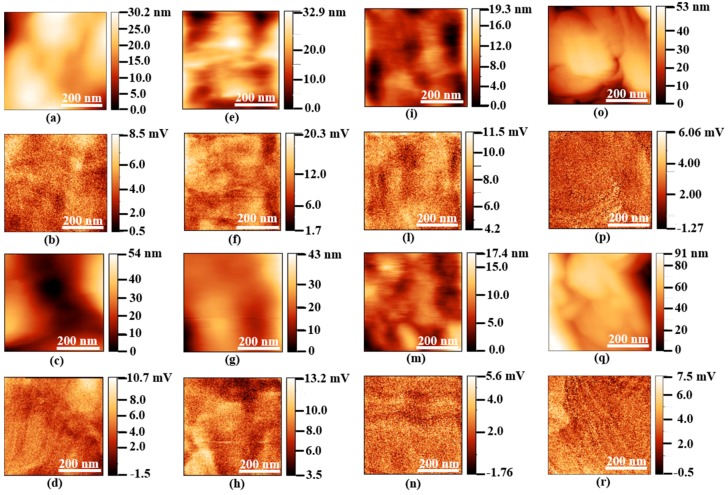
Morphological maps and vertical (out-of-plane) PFM signals at *V_ac_* = 10 V and at 15 kHz for Neat-PVDF (**a**,**b**), for M1-HS1 (**c**,**d**), for M1-HS2 (**e**,**f**), for M1-HS3 (**g**,**h**), for M1-HS4 (**i**,**l**), for M1-HS5 (**m**,**n**), for M2-HS1-GNP (**o**,**p**), and for M2-HS1-ZnO (**q**,**r**).

Firstly, we report in Figure 7 the topographic maps and the amplitude of the vertical (out-of-plane) signal measured through PFM over a scan area (500 × 500) nm^2^ in size of the produced samples. The scanning areas were limited to a lateral size of only 500 nm in order to avoid cross-talk between the amplitude of the vertical PFM and the topographic signals. In fact, the PFM scanned area must be much smaller than the average size of spherulites, which ranges from about 10 μm to about 35 μm, as reported in Table 2. Figure 7 shows that there is not a direct correlation between the amplitude of the vertical PFM signal and the AFM signal due to the small size of the investigated area with respect to the size of the spherulites.

**Figure 8:**
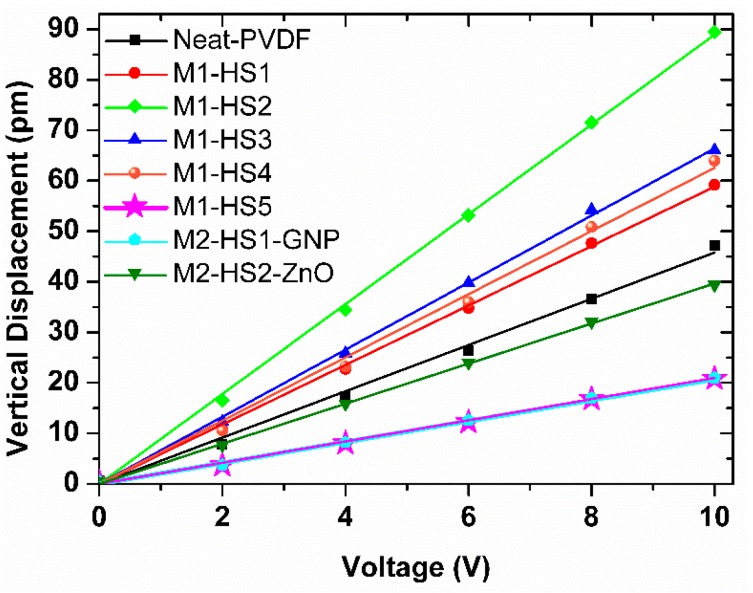
Average amplitude of the vertical displacement measured through PFM as a function of the applied voltage $V_{ac}$.

Next, we evaluated the average amplitude of the measured vertical displacement for each sample as a function of the amplitude (*V_ac_*) of the applied alternating voltage. The obtained data, which are averaged over 10 scanning areas, and the corresponding linear fits are reported in Figure 8. The slope of the straight lines interpolating the measured data is the piezoelectric coefficient *d*_33_.

**Figure 9:**
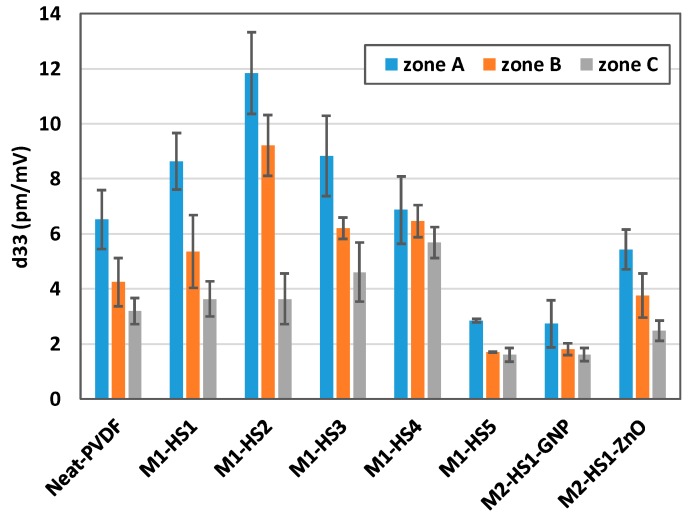
Measured average piezoresponse coefficient in the three zones of the produced samples, with standard deviation.

The average piezoelectric coefficients *d*_33_ of the three different zones A, B, and C sketched in Figure 2 for each sample are reported in Figure 9, together with the corresponding standard deviations. We notice that the highest piezoelectric coefficient is provided by sample M1-HS2, produced using magnesium nitrate hexahydrate salt, with the maximum value of 13.48 pm/mV in zone A, located over a spherulite. The worst piezoelectric performances are observed in sample M1-HS5, made with iron nitrate hexahydrate salt, and in the samples combining HMS-dissolution and nanofiller dispersion. In all cases, the standard deviation of the measured *d*_33_ in the different zones of the produced samples reaches maximum values of 24.6%, 25%, and 23.4% in the samples that are characterized by the highest roughness of the spherulite surface, as resulting from the AFM scanning in Figure 7. This proves a correlation between piezoresponse and the local nanoscale morphology of the sample surface.

We computed the global average value of the piezoresponse coefficient $\langle d_{33}\rangle$ for each sample, according to Equation (6). The obtained values, including the corresponding standard deviations, are reported in Table 2. It is confirmed that the highest piezoresponse is observed in sample M1-HS2. In this case, the standard deviation varies in the range 30–40% since it is representative of the local variation of *d*_33_ over the sample surface.

**Figure 10:**
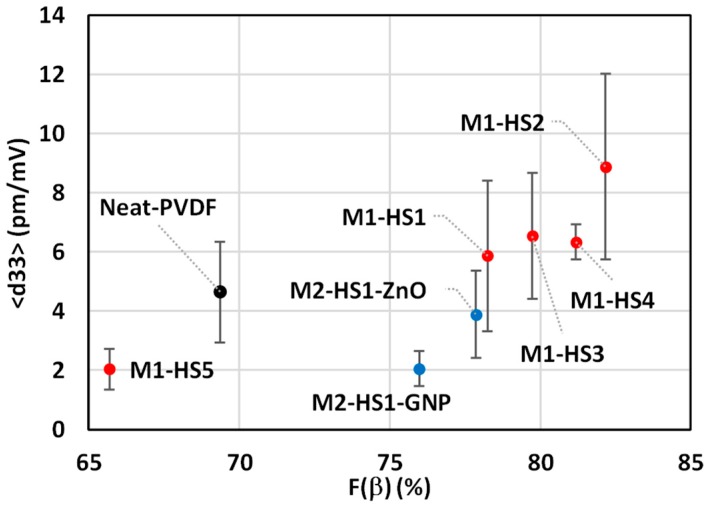
Averaged *d_33_* as a function of F(β) of all produced samples, with standard deviation.

In addition, Figure 10 shows $\langle d_{33}\rangle$ as a function of F(β) [23]. As expected from FT-IR and XRPD data, we notice that when we dissolve the HMS into PVDF, the piezoelectric coefficient increases. Indeed, the maximum value of $\langle d_{33}\rangle$ = (8.88 ± 3.145) pm/V was observed in sample M1-HS2, which is also characterized by the highest value of F(β), while the lowest value of $\langle d_{33}\rangle$ = (2.047 ± 0.69) pm/V was observed in sample M1-HS5, in agreement with the FT-IR and XRPD data showing the lowest *β*-phase fraction.

Moreover, when we added both HMS and nanofillers, in contrast with the observed increase of the *β*-phase deduced from the FT-IR and XRPD data, we found a decrease of $\langle d_{33}\rangle$ compared with the neat PVDF.

**Figure 11:**
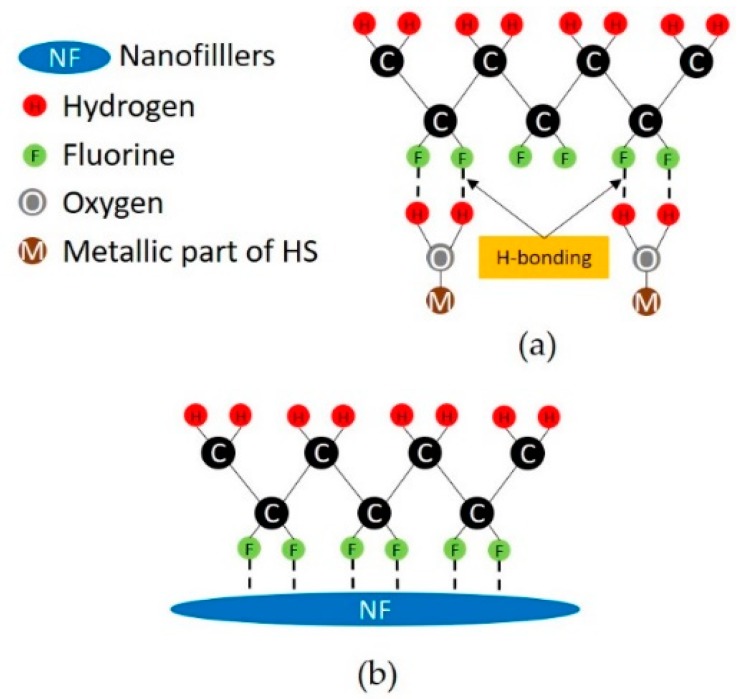
Schematic representation of (**a**) the formation of H-bonding with the metallic part of HS and the CF_2_ group of PVDF, and (**b**) the electrostatic interaction between the nanofillers (either GNP or ZnO NRs) and the CF_2_ group of PVDF.

We believe this behavior is due to a destructive interaction between the dissolved HMS and nanofillers (either GNPs or ZnO NRs). Dissolved HMS tends to form hydrogen bonding with the CF_2_ group of the PVDF, which promotes electrostatic interactions between the PVDF polymer chain and metallic salts in the polar solvent, as sketched in Figure 11 [26,28]. Moreover, we notice the formation of some nanofiller agglomerations in samples including either GNPs or ZnO NRs. For instance, Figure 4a clearly shows the presence of GNP agglomerations over the sample surface, which interfere with the formation of spherulites and, in turn, with the enhancement of the *β*-phase. Actually, the presence of filler agglomerates is observed in the sample with the lowest value of piezoresponse coefficient (i.e., M2-HS1-GNP).

**Figure 12:**
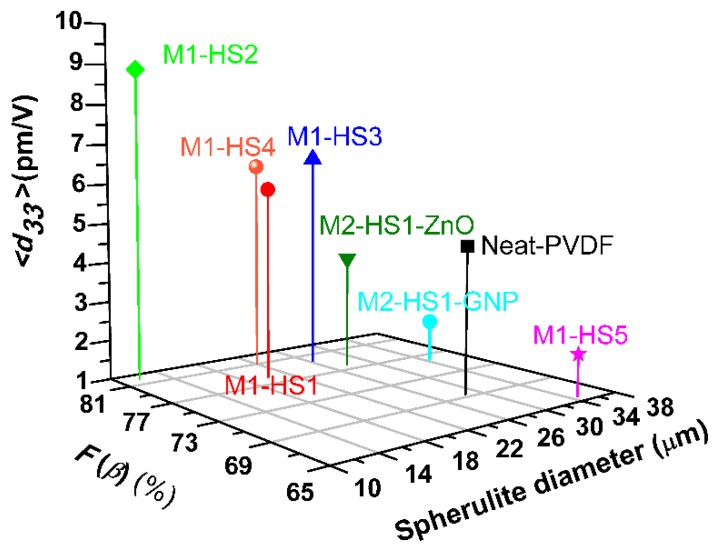
Averaged piezoelectric coefficient, *d_33_*, vs relative fraction of the *β*-phase, F(β), and averaged spherulite diameter of the produced samples.

The incoherent interaction between HMS and nanofiller induces an incoherent distribution of the *β*-phase polymer chains, resulting in a poor *d*_33_ signal. Furthermore, we observe that in most samples, when the average value of the spherulite diameter decreases, the piezoelectric coefficient of the PVDF nanocomposite films increases (Figure 12). This trend is also observed with respect to the fraction of the *β*-phase, F(β).

Accordingly, the minimum value of *d_33_* and the largest spherulite diameter are observed in the sample containing the Fe-HMS (i.e., M1-HS5), due to the relatively large mass and small negative value of the standard electrode potential of Fe^3+^, which weaken the hydrogen bond with PVDF. On the contrary, the highest value of *d_33_* (combined with the smaller size of spherulites) is observed in the sample containing the Mg-HMS (i.e., M1-HS2), with the standard electrode potential of the Mg^2+^ ion being the most negative among the metals in the other HMSs used. These results are in agreement with data reported in literature [37,38], in which it is shown that a smaller diameter of the spherulites corresponds to a higher presence of *β*-phase and, consequently, to a higher piezoelectric coefficient.

Finally, the sample M1-HS2 was characterized using a mini-shaker after being contacted with gold over the top and bottom surfaces. In this case, the measured $d_{33}$ was 9.00 pm/mV, which is in good agreement with the value of $\langle d_{33}\rangle$ = 8.88 pm/mV reported in Table 2, and resulting from the average response over the three considered zones of the sample, as sketched in Figure 2.

## 4. Conclusions

Free-standing flexible PVDF nanocomposite films with a dominant HMS-induced electroactive phase were successfully prepared through a simple, cost-effective, time-saving solution-casting method without electrical poling.

Two different production routes were investigated to induce this electroactive phase, based on dissolution of HMS in the polymer or on nanofiller dispersion in combination with HMS-dissolution. FT-IR and XRPD investigations revealed that the incorporation of Zn(NO_3_)_2_·6H_2_O, Mg(NO_3_)_2_·6H_2_O, MgCl_2_·6H_2_O, and AlCl_3_·6H_2_O salts into the PVDF matrix induces an increase of the electroactive phase, which can be ascribed to the combined effect of the change in the inherent dipole moment of the electroactive phase contained in the PVDF itself, and of the formation of H-bonding between the metallic part of the HMS filler and of PVDF via electrostatic interactions [28]. This combined effect is enhanced in the PVDF nanocomposite produced using HMS containing Mg nitrate, since Mg^2+^ is characterized by the most negative redox potential with respect to the other metal ions considered in this study. This result is in line with the finding that the sample produced through dissolution in PVDF of FeCl_3_·6H_2_O, in which the ion Fe^3+^ has a nearly zero standard potential, has a very poor piezoelectric response.

The increase of the *β*-phase fraction in the samples M1-HS1, M1-HS2, M1-HS3, and M1-HS4, produced through dissolution of an HMS containing metals with negative redox potential, was correlated to the enhancement of the piezoelectric coefficient, measured through PFM. At the same time, the reduction of *β*-phase in the sample M1-HS5 containing Fe corresponds to the reduction of the piezoelectric coefficient compared with neat PVDF. In particular, the highest average value of *d_33_* (i.e., 8.88 ± 3.14 pm/V) and the highest local peak value (i.e., 13.49 pm/V) were measured in the sample containing Mg(NO_3_)_2_·6H_2_O salt (M1-HS2). This sample also contained the highest fraction of *β*-phase (i.e., 82.18%) with respect to all other samples, and was characterized by the lowest average value of spherulite diameter (i.e., 11.87 ± 3.74 μm).

Another finding of these study is that although XRPD and FT-IR measurements show that samples M2-HS1-GNP and M2-HS1-ZnO present a higher fraction of *β*-phase than does the neat sample, PFM measurements showed an average *d_33_* lower than that of the neat sample. We speculate that the reason for this should be connected with a poor alignment of the electroactive polymer chains along the vertical axis due to a destructive electrostatic interaction between the metallic part of the HMS filler, the nanofillers (GNP and ZnO NRs), and the CF_2_ group of the PVDF. Moreover, from AFM topological analysis of the produced samples, we speculate that the microstructure of their surface has some influence on the piezoelectric response. A definitely negative effect on the piezoresponse coefficient of the samples produced by combining HMS-dissolution and nanofiller dispersion is observed in the case of agglomerate formation.

In any case, it is worth underlining that if we analyze the samples produced through the production process M1 or M2, we observe that the piezoelectric coefficient increases as the relative fraction of *β*-phase rises. Moreover, *d_33_* and the fraction of *β*-phase in general increase as the dimension of the averaged spherulite diameter decreases.

Compared with our previous studies, the addition of HMS salts results in a marked improvement of the *d_33_* value [22,23,26]. In particular, we are able to enhance the piezoelectric coefficient of modified PVDF films with obtained values consistent with those reported in the literature [9,17,39], but through a facile, cost-effective, and time-saving production route.

Our XRPD and FT-IR findings were corroborated by SEM investigation, revealing that the nucleation kinetics are enhanced by the presence of the HMS salts, as evidenced by the formation of an increasing number of spherulites with increasing numbers of nucleation sites, in turn leading to a reduction of the average spherulite diameter.

This result opens new routes to the possibility of producing electroactive polymers with tailored electroactive properties and resonant frequency, through the control of the effective piezoelectric properties of the material, which is achieved by means of nanofiller dispersion into the polymer matrix.

## Figures and Tables

**Table 1:** List of the produced specimens of PVDF nanocomposite adding graphene nanoplatelets (GNPs), zinc oxide nanoroads (ZnO-NRs) and hexahydrate metallic salt (HMS).

Sample	Production Method	GNPs (wt %)	ZnO NRs (wt %)	HMS (0.2 wt %)
Neat-PVDF	-	-	-	-
M1-HS1	M1	-	-	Zn(NO_3_)_2_·6H_2_O
M1-HS2	M1	-	-	Mg(NO_3_)_2_·6H_2_O
M1-HS3	M1	-	-	MgCl_2_·6H_2_O
M1-HS4	M1	-	-	AlCl_3_·6H_2_O
M1-HS5	M1	-	-	FeCl_3_·6H_2_O
M2-HS1-GNP	M2	0.1	-	Zn(NO_3_)_2_·6H_2_O
M2-HS1-ZnO	M2	-	0.1	Zn(NO_3_)_2_·6H_2_O

**Table 2:** List of the produced PVDF films, including the type of HMS dissolved; the average values of the spherulite diameter estimated from FE-SEM images; the relative fraction of the *β*-phase, F(*β*), estimated from FT-IR spectra; and the average piezoelectric coefficient 〈*d_33_*〉 obtained through PFM measurements.

Sample	HMS Type (0.2 wt %)	Spherulite Diameter (µm)	F(β) (%)	$\langle d_{33}\rangle$ (pm/V)
Neat-PVDF	-	28.58 ± 4.56	69.36	4.65± 1.70
M1-HS1	Zn(NO_3_)_2_·6H_2_O	20.09 ± 5.33	78.25	5.87 ± 2.54
M1-HS2	Mg(NO_3_)_2_·6H_2_O	11.87 ± 3.74	82.17	8.88 ± 3.14
M1-HS3	MgCl_2_·6H_2_O	26.37 ± 5.17	79.73	6.54 ± 2.13
M1-HS4	AlCl_3_·6H_2_O	22.19 ± 4.59	81.18	6.34 ± 0.60
M1-HS5	FeCl_3_·6H_2_O	34.84 ± 4.36	65.70	2.04 ± 0.69
M2-HS1-GNP	Zn(NO_3_)_2_·6H_2_O	34.55 ± 4.67	75.98	2.05 ± 0.60
M2-HS1-ZnO	Zn(NO_3_)_2_·6H_2_O	27.79 ± 5.50	77.87	3.89 ± 1.48
